# Supplementary material for: CeGAL: Redefining a Widespread Fungal-Specific Transcription Factor Family Using an In Silico Error-Tracking Approach
Source: J Fungi (Basel). 2023 Mar 29;9(4):424. doi: 10.3390/jof9040424 (PMC10141177; doi:10.3390/jof9040424)
Supplement: Supplementary file 1 [file jof-09-00424-s001.zip › Supplementary Methods.pdf]

Supplementary methods

Assessment of over-prediction

In order to determine whether our sequence curation protocol over-predicts DBDs in the potentially erroneous sequences, we used our error identification protocol to search for Zn2C6 DBD in the C-terminal region of the proteins (*i.e.* downstream of the MHD). Out of the 80456 proteins annotated with Zn2C6 and MHD in the UniProt database, there exists a small number of 76 proteins (0.1%) where the domain order is inversed with respect to the canonical DBD-MHD architecture. According to the InterPro database, 16 different domain architectures are found with the DBD located downstream of the MHD, where the most frequent architectures are 23 proteins with Zn2C6-MHD-Zn2C6-MHD, 15 proteins with MHD-Zn2C6-MHD, 15 proteins with Zn2C6-MHD-Zn2C6, and 10 proteins with MHD-Zn2C6. It is worth noting that all 76 proteins are from the unreviewed TrEMBL database and none are from the biocurated Swiss-Prot database.

Using the same set of 16760 potentially erroneous sequences, we searched for Zn2C6 DBD downstream of the MHD extending the gene 1000 nt downstream of the defined 3' end. A total of 110 DBD (0.6%) were found in this extended C-terminal region. A manual analysis of the 110 genes showed that for 84 sequences (75%), the downstream gene coded for a protein annotated with either a Zn2C6 or a MHD. For a further 6 proteins, the downstream gene coded for other domains already observed in combination with a Zn2C6 in the public databases. Of the remaining 20 sequences, 17 also had N-terminal Zn2C6 domain hits and thus correspond to Zn2C6-MHD-Zn2C6 type architectures.

Transcriptome analysis of mispredicted genes in *S. cerevisiae* strains

The NCBI Gene Expression Omnibus (GEO) project (Barrett et al., 2013) was searched for RNA-seq datasets concerning the *S. cerevisiae* strains with at least 10 potentially mispredicted genes: AWRI796, FostersB, FostersO, LalvinQA23, Vin13, and VL3. No datasets were found for FostersB, FostersO, LalvinQA23 and Vin13, and we therefore focused on the GSE57282 dataset that included expression data for the strains AWRI96 (SRR1269766) and VL3 (SRR1269765). After downloading and decompressing the dataset, the integrity of the data was checked and Trim Galore (v0.6.1) (DOI: 10.5281/zenodo.7598955) was used for quality and adapter trimming as well as a quality control check. The AWRI96 and VL3 genomes were downloaded from the Saccharomyces Genome Database (SGD) (Wong et al., 2023) as FASTA files, together with the gene annotations as GFF3 files. Using the STAR program (2.7.10a), the reads of the trimmed FASTQ file were aligned against the genome FASTA file and annotation GFF3 file of the corresponding strain. For each mispredicted gene with a coverage of at least 30 reads, the BAM files generated by STAR were then manually reviewed with the Integrative Genome Viewer (IGV) browser (Thorvaldsdóttir et al., 2013) to explore the read coverage around the gene. Figure S1 shows the results for the seven AWRI96 genes and the five VL3 genes with sufficient coverage in the GEO dataset.

Barrett T, Wilhite SE, Ledoux P, Evangelista C, Kim IF, Tomashevsky M, Marshall KA, Phillippy KH, Sherman PM, Holko M, Yefanov A, Lee H, Zhang N, Robertson CL, Serova N, Davis S, Soboleva A. NCBI GEO: archive for functional genomics data sets--update. Nucleic Acids Res. 2013 41:D991-5.

Wong ED, Miyasato SR, Aleksander S, Karra K, Nash RS, Skrzypek MS, Weng S, Engel SR, Cherry JM. Saccharomyces Genome Database Update: Server Architecture, Pan-Genome Nomenclature, and External Resources. Genetics. 2023 iyac191.

Thorvaldsdóttir H, Robinson JT, Mesirov JP. Integrative Genomics Viewer (IGV): high-performance genomics data visualization and exploration. Brief Bioinform. 2013 14:178-92.
