# Supplementary material for: CeGAL: Redefining a Widespread Fungal-Specific Transcription Factor Family Using an In Silico Error-Tracking Approach
Source: J Fungi (Basel). 2023 Mar 29;9(4):424. doi: 10.3390/jof9040424 (PMC10141177; doi:10.3390/jof9040424)
Supplement: Supplementary file 1 [file jof-09-00424-s001.zip › Table S1.pdf]

Table S1. List of the 44 Zn2C6-MHD containing genes coded by the Saccharomyces cerevisiae S288C genome.

UniprotKB ID	Gene ID	SGD ID
Q04052	ARO80	YDR421W
P40467	ASG1	YIL130W
P39113	CAT8	YMR280C
P40969	CEP3	YMR168C
P43634	CHA4	YLR098C
P21657	DAL81	YIR023W
P38073	EDS1	YBR033W
P04386	GAL4	YPL248C
Q12180	HAL9	YOL089C
P0CE41	HAP1	YLR256W
P08638	LEU3	YLR451W
P53338	MAL13	YGR288W
P38157	MAL33	YBR297W
P39720	OAF1	YAL051W
P36023	OAF3	YKR064W
P12383	PDR1	YGL013C
P33200	PDR3	YBL005W
Q06149	PDR8	YLR266C
P52960	PIP2	YOR363C
P07272	PPR1	YLR014C
P53749	PUL4	YNR063W
P25502	PUT3	YKL015W
Q08904	RDR1	YOR380W
P25611	RDS1	YCR106W
P32862	RGT1	YKL038W
Q06639	RSC3	YDR303C
P38781	RSC30	YHR056C
P34228	SEF1	YBL066C
P46954	SIP4	YJL089W
P50104	STB4	YMR019W
P38699	STB5	YHR178W
P38114	TBS1	YBR150C
P47988	TEA1	YOR337W
P39961	TOG1	YER184C
Q04411	URC2	YDR520C

Q03631	WAR1	YML076C
Q12340	YRM1	YOR172W
Q12172	YRR1	YOR162C
P43551	ZNF1	YFL052W
P35995	YKW2	YKL222C
P39529	YJU6	YJL206C
Q05854	YL278	YLR278C
Q06595	YP196	YPR196W
Q12244	YL054	YLL054C
