# Supplementary material for: CeGAL: Redefining a Widespread Fungal-Specific Transcription Factor Family Using an In Silico Error-Tracking Approach
Source: J Fungi (Basel). 2023 Mar 29;9(4):424. doi: 10.3390/jof9040424 (PMC10141177; doi:10.3390/jof9040424)
Supplement: Supplementary file 1 [file jof-09-00424-s001.zip › Table S2.pdf]

*Table S2. List of the 47 genomes of Saccharomyces cerevisiae strains extracted from the SGD.*

| Strain | No. of scaffolds | No. of ORFs | Description |
|---|---|---|---|
| **AWRI1631** | **2484** | 4747 | Haploid derivative of South African commercial wine strain N96 |
| **AWRI796** | **105** | 4747 | South African red wine strain |
| **BC187** | **614** | 5254 | Derivative of California wine barrel isolate |
| **BY4741 (Stanford)** | **864** | 6682 | S288C-derivative laboratory strain |
| **BY4742 (Stanford)** | **868** | 5391 | S288C-derivative laboratory strain |
| **CBS7960** | **2367** | 5333 | Brazilian bioethanol factory isolate |
| **CEN.PK113 -7D** | **589** | 6269 | Lab strain constructed by Michael Ciriacy and K.D. Entian |
| **CLIB215** | **1566** | 5051 | New Zealand bakery isolate |
| **CLIB324** | **3528** | 3733 | Vietnamese bakery isolate |
| **D273-10B** | **203** | 5367 | Lab strain for mitochondrial studies constructed in the Sherman lab |
| **DBVPG6044** | **624** | 5297 | West African isolate |
| **EC1118** | **31** | 6352 | Commercial wine strain |
| **EC9-8** | **1088** | 5490 | Haploid derivative of Israeli canyon isolate |
| **FL100 (SGD)** | **174** | 5364 | lab strain constructed by Francis Lacroute |
| **FostersB** | **142** | 6240 | Commercial ale strain |
| **FostersO** | **222** | 6239 | Commercial ale strain |
| **FY1679** | **388** | 5370 | S288C-derivative laboratory strain |
| **JAY291** | **453** | 6143 | Haploid derivative of Brazilian industrial bioethanol strain PE-2 |
| **JK9-3d** | **197** | 5358 | Lab strain constructed by J. Kunz and J. Heitman while in the Hall lab |
| **K11** | **480** | 5267 | Sake strain |
| **Kyokai7** | **705** | 6285 | Japanese sake yeast |
| **L1528** | **538** | 5361 | Chilean wine strain |
| **LalvinQA23** | **185** | 6293 | Portuguese Vinho Verde white wine strain |
| **M22** | **6145** | 2548 | Italian vineyard isolate |
| **PW5** | **1608** | 5523 | Nigerian Raphia palm wine isolate |
| **RedStar** | **577** | 5440 | Commercial baking strain |
| **RM11-1A (SGD)** | **115** | 5310 | Natural isolate collected by Robert Mortimer from a Calif vineyard |

| | | | |
|---|---|---|---|
| **S288C** | **17** | 6716 | Reference Genome |
| **SEY6210** | **183** | 5383 | Lab strain synonymous with SEY6211, Mata haploid, diploid SEY6210.1 |
| **Sigma1278b -10560-6B (SGD)** | **214** | 5343 | Used to study pseudohyphal growth isolated in Grenson lab in 1960s |
| **SK1** | **106** | 5960 | High sporulating diploid lab strain used to study sporulation, meiosis |
| **T7** | **360** | 6293 | Missouri oak tree exudate isolate |
| **T73** | **5882** | 3324 | Spanish red wine strain |
| **UC5** | **1527** | 5599 | Japanese sake yeast |
| **UWOPS05-217-3** | **1348** | 4788 | Environmental isolate |
| **Vin13** | **158** | 6321 | South African white wine strain |
| **VL3** | **125** | 6322 | French white wine strain |
| **W303 (SGD)** | **236** | 5357 | Lab strain constructed by Rodney Rothstein |
| **X2180-1A** | **212** | 5370 | Lab strain constructed from Robert Mortimer |
| **Y10** | **4118** | 2656 | Philippine coconut isolate |
| **Y55** | **198** | 5330 | Lab strain originally isolated from wine grapes in France by O. Winge |
| **YJM269** | **1179** | 5396 | Austrian Blauer Portugieser wine grape isolate |
| **YJM339** | **554** | 5317 | Clinical isolate |
| **YJM789** | **258** | 6378 | Haploid derivative of opportunistic human pathogen |
| **YPS128** | **670** | 5241 | Pennsylvania woodland isolate |
| **YPS163 (Stanford)** | **607** | 5263 | Pennsylvania woodland isolate |
| **YS9** | **851** | 5271 | Singapore baking strain |
| **ZTW1** | **33** | 6306 | Chinese corn mash bioethanol isolate |
