# Supplementary material for: CeGAL: Redefining a Widespread Fungal-Specific Transcription Factor Family Using an In Silico Error-Tracking Approach
Source: J Fungi (Basel). 2023 Mar 29;9(4):424. doi: 10.3390/jof9040424 (PMC10141177; doi:10.3390/jof9040424)
Supplement: Supplementary file 1 [file jof-09-00424-s001.zip › Table S3.pdf]

*Table S3.* Results of the analysis of 253 MHD-containing sequences in the SGD database.

| SGD sequence identifier | Source of error | C6 motif | hmmsearch E-value |
|---|---|---|---|
| YAL051W_FostersB | FRAMESHIFT | FULL | 7.0e-16 |
| YAL051W_FostersO | FRAMESHIFT | FULL | 7.0e-16 |
| YAL051W_LalvinQA23 | FRAMESHIFT | FULL | 7.0e-16 |
| YAL051W_Vin13 | FRAMESHIFT | FULL | 7.0e-16 |
| YAL051W_VL3 | FRAMESHIFT | FULL | 7.0e-16 |
| YBL005W_AWRI796 | FRAMESHIFT | FULL | 8.2e-13 |
| YBL005W_FostersO | FRAMESHIFT | FULL | 8.5e-13 |
| YBL005W_YJM269 | FRAMESHIFT | FULL | 8.4e-13 |
| YBL066C_AWRI796 | FRAMESHIFT | FULL | 2.8e-15 |
| YBL066C_FostersO | FRAMESHIFT | FULL | 6.5e-15 |
| YBL066C_LalvinQA23 | FRAMESHIFT | FULL | 2.8e-15 |
| YBL066C_Vin13 | FRAMESHIFT | FULL | 2.8e-15 |
| YBL066C_YJM269 | FRAMESHIFT | FULL | 2.8e-15 |
| YBR033W_AWRI796 | FRAMESHIFT | FULL | 6.4e-13 |
| YBR033W_CBS7960 | START CODON | FULL | 6.6e-13 |
| YBR033W_FostersB | FRAMESHIFT | FULL | 6.5e-13 |
| YBR033W_K11 | START CODON | FULL | 6.7e-13 |
| YBR033W_Kyokai7 | START CODON | FULL | 6.7e-13 |
| YBR033W_RedStar | START CODON | FULL | 6.6e-13 |
| YBR033W_T7 | START CODON | FULL | 6.2e-13 |
| YBR033W_UC5 | START CODON | FULL | 6.7e-13 |
| YBR033W_VL3 | FRAMESHIFT | FULL | 6.5e-13 |
| YBR033W_YPS128 | START CODON | FULL | 6.6e-13 |
| YBR033W_YPS163 | START CODON | FULL | 6.6e-13 |
| YBR033W_YS9 | START CODON | FULL | 6.5e-13 |
| YBR033W_ZTW1 | FRAMESHIFT | FULL | 6.6e-13 |
| YBR150C_LalvinQA23 | FRAMESHIFT | FULL | 1.3e-12 |
| YBR150C_Vin13 | FRAMESHIFT | FULL | 1.3e-12 |
| YBR150C_VL3 | FRAMESHIFT | FULL | 1.2e-12 |
| YBR297W_AWRI1631 | FRAMESHIFT | FULL | 2.3e-17 |
| YBR297W_AWRI796 | FRAMESHIFT | FULL | 2.4e-17 |
| YBR297W_BC187 | SCAFFOLD | FULL | 2.3e-17 |
| YBR297W_FY1679 | Unknown | FULL | 3.0e-17 |
| YBR297W_Vin13 | SCAFFOLD | FULL | 5.1e-17 |
| YBR297W_W303 | Unknown | FULL | 3.0e-17 |
| YBR297W_X2180-1A | Unknown | FULL | 3.0e-17 |
| YCR106W_AWRI796 | FRAMESHIFT | FULL | 1.4e-13 |
| YCR106W_BY4741 | SCAFFOLD | NONE | 1.0e+03 |

| | | | |
|---|---|---|---|
| YCR106W_BY4742 | Undetermined genome region | FULL | 1.4e-13 |
| YCR106W_CBS7960 | REPEAT | FULL | 1.4e-13 |
| YCR106W_FY1679 | FRAMESHIFT | FULL | 7.2e-09 |
| YCR106W_LalvinQA23 | FRAMESHIFT | FULL | 1.4e-13 |
| YCR106W_M22 | FRAMESHIFT | FULL | 1.4e-13 |
| YCR106W_Sigma1278b | SCAFFOLD | NONE | 1.0e+03 |
| YCR106W_Vin13 | FRAMESHIFT | FULL | 1.4e-13 |
| YCR106W_VL3 | FRAMESHIFT | FULL | 1.4e-13 |
| YCR106W_W303 | SCAFFOLD | NONE | 1.0e+03 |
| YCR106W_X2180-1A | SCAFFOLD | NONE | 1.0e+03 |
| YCR106W_YPH499 | Undetermined genome region | FULL | 1.4e-13 |
| YCR106W_YPS128 | SCAFFOLD | PARTIAL | 2.0e-07 |
| YCR106W_YPS163 | SCAFFOLD | PARTIAL | 4.4e-07 |
| YDR303C_EC1118 | FRAMESHIFT | FULL | 1.1e-12 |
| YDR303C_FostersB | FRAMESHIFT | FULL | 1.1e-12 |
| YDR303C_FostersO | FRAMESHIFT | FULL | 1.1e-12 |
| YDR303C_Vin13 | START CODON | FULL | 1.1e-12 |
| YDR303C_VL3 | FRAMESHIFT | FULL | 4.3e-12 |
| YDR421W_FostersB | FRAMESHIFT | FULL | 9.0e-12 |
| YDR421W_FostersO | FRAMESHIFT | FULL | 9.1e-12 |
| YDR421W_JAY291 | START CODON | FULL | 8.9e-12 |
| YDR520C_AWRI796 | FRAMESHIFT | FULL | 8.3e-10 |
| YDR520C_CLIB324 | FRAMESHIFT | FULL | 8.3e-10 |
| YDR520C_FostersB | FRAMESHIFT | FULL | 8.2e-10 |
| YER184C_AWRI796 | FRAMESHIFT | FULL | 1.9e-13 |
| YER184C_FostersB | FRAMESHIFT | FULL | 2.0e-13 |
| YER184C_FostersO | FRAMESHIFT | FULL | 2.9e-13 |
| YER184C_LalvinQA23 | FRAMESHIFT | FULL | 1.9e-13 |
| YER184C_Vin13 | FRAMESHIFT | PARTIAL | 3.2e-11 |
| YER184C_ZTW1 | FRAMESHIFT | FULL | 2.0e-13 |
| YGL013C_CLIB215 | FRAMESHIFT | FULL | 4.7e-14 |
| YGL013C_FostersB | FRAMESHIFT | FULL | 4.7e-14 |
| YGL013C_FostersO | FRAMESHIFT | FULL | 4.7e-14 |
| YGL013C_Vin13 | FRAMESHIFT | FULL | 4.7e-14 |
| YGR288W_FostersB | START CODON | FULL | 2.1e-14 |
| YGR288W_FostersO | START CODON | FULL | 2.1e-14 |
| YGR288W_VL3 | START CODON | FULL | 2.2e-14 |
| YHR056C_FostersB | FRAMESHIFT | FULL | 3.3e-12 |
| YHR056C_M22 | SCAFFOLD BORDER | NONE | 1.0e+03 |
| YHR056C_RM11-1a | START CODON | FULL | 3.6e-12 |
| YHR056C_T73 | FRAMESHIFT | FULL | 1.2e-12 |

| | | | |
|---|---|---|---|
| YHR056C_VL3 | FRAMESHIFT | FULL | 3.6e-12 |
| YHR178W_FostersO | FRAMESHIFT | FULL | 7.1e-13 |
| YHR178W_Vin13 | FRAMESHIFT | FULL | 7.1e-13 |
| YHR178W_VL3 | FRAMESHIFT | FULL | 7.1e-13 |
| YHR178W_ZTW1 | FRAMESHIFT | FULL | 7.1e-13 |
| YIL130W_AWRI796 | FRAMESHIFT | FULL | 3.9e-15 |
| YIL130W_FostersB | FRAMESHIFT | FULL | 5.1e-13 |
| YIL130W_FostersO | FRAMESHIFT | FULL | 5.1e-13 |
| YIL130W_LalvinQA23 | FRAMESHIFT | FULL | 3.9e-15 |
| YIL130W_Vin13 | FRAMESHIFT | FULL | 3.9e-15 |
| YIL130W_VL3 | FRAMESHIFT | FULL | 3.9e-15 |
| YIL130W_Y55 | FRAMESHIFT | FULL | 3.9e-15 |
| YIR023W_AWRI796 | FRAMESHIFT | FULL | 4.7e-12 |
| YIR023W_EC1118 | FRAMESHIFT | FULL | 4.7e-12 |
| YIR023W_FostersB | FRAMESHIFT | FULL | 4.8e-12 |
| YIR023W_FostersO | FRAMESHIFT | FULL | 1.2e-11 |
| YIR023W_LalvinQA23 | FRAMESHIFT | FULL | 4.5e-12 |
| YIR023W_T73 | FRAMESHIFT | FULL | 4.7e-12 |
| YIR023W_VL3 | FRAMESHIFT | FULL | 4.5e-12 |
| YIR023W_YJM269 | FRAMESHIFT | FULL | 4.7e-12 |
| YJL089W_AWRI796 | FRAMESHIFT | FULL | 2.6e-15 |
| YJL089W_FostersB | FRAMESHIFT | FULL | 2.6e-15 |
| YJL089W_FostersO | FRAMESHIFT | FULL | 2.6e-15 |
| YJL089W_LalvinQA23 | FRAMESHIFT | FULL | 2.6e-15 |
| YJL089W_VL3 | FRAMESHIFT | FULL | 2.6e-15 |
| YJL206C_AWRI796 | FRAMESHIFT | FULL | 1.0e-13 |
| YJL206C_CBS7960 | FRAMESHIFT | FULL | 1.0e-13 |
| YJL206C_CLIB215 | FRAMESHIFT | FULL | 1.0e-13 |
| YJL206C_FostersB | FRAMESHIFT | FULL | 1.1e-13 |
| YJL206C_Sigma1278b | START CODON | FULL | 1.0e-13 |
| YJL206C_Vin13 | FRAMESHIFT | FULL | 1.0e-13 |
| YKL015W_AWRI796 | FRAMESHIFT | FULL | 1.1e-14 |
| YKL015W_FostersB | FRAMESHIFT | FULL | 1.1e-14 |
| YKL015W_FostersO | FRAMESHIFT | FULL | 7.9e-14 |
| YKL015W_LalvinQA23 | FRAMESHIFT | FULL | 1.1e-14 |
| YKL015W_T73 | FRAMESHIFT | FULL | 1.0e-14 |
| YKL015W_Vin13 | FRAMESHIFT | FULL | 2.2e-14 |
| YKL015W_VL3 | FRAMESHIFT | FULL | 2.2e-14 |
| YKL015W_YJM269 | FRAMESHIFT | FULL | 1.1e-14 |
| YKL038W_D273-10B | START CODON | FULL | 5.3e-14 |
| YKL038W_DBVPG6044 | START CODON | FULL | 5.3e-14 |
| YKL038W_EC1118 | FRAMESHIFT | FULL | 5.3e-14 |
| YKL038W_EC9-8 | FRAMESHIFT | FULL | 5.3e-14 |

| | | | |
|---|---|---|---|
| YKL038W_FostersB | START CODON | FULL | 5.3e-14 |
| YKL038W_FostersO | FRAMESHIFT | FULL | 5.3e-14 |
| YKL038W_K11 | START CODON | FULL | 5.3e-14 |
| YKL038W_Kyokai7 | START CODON | FULL | 5.3e-14 |
| YKL038W_LalvinQA23 | FRAMESHIFT | FULL | 5.3e-14 |
| YKL038W_PW5 | START CODON | FULL | 5.3e-14 |
| YKL038W_RedStar | START CODON | FULL | 5.2e-14 |
| YKL038W_Sigma1278b | START CODON | FULL | 5.3e-14 |
| YKL038W_SK1 | START CODON | FULL | 5.3e-14 |
| YKL038W_T7 | START CODON | FULL | 5.3e-14 |
| YKL038W_UC5 | FRAMESHIFT | FULL | 5.3e-14 |
| YKL038W_Vin13 | FRAMESHIFT | FULL | 5.3e-14 |
| YKL038W_VL3 | FRAMESHIFT | FULL | 5.3e-14 |
| YKL038W_Y55 | START CODON | FULL | 5.3e-14 |
| YKL038W_YJM339 | START CODON | FULL | 5.4e-14 |
| YKL038W_YJM789 | START CODON | FULL | 5.3e-14 |
| YKL038W_YS9 | START CODON | FULL | 5.3e-14 |
| YKL038W_ZTW1 | START CODON | FULL | 5.3e-14 |
| YKL222C_AWRI1631 | FRAMESHIFT | FULL | 3.9e-10 |
| YKL222C_CBS7960 | FRAMESHIFT | FULL | 3.9e-10 |
| YKL222C_EC9-8 | START CODON | FULL | 3.9e-10 |
| YKL222C_FostersB | FRAMESHIFT | FULL | 2.0e-10 |
| YKL222C_FostersO | FRAMESHIFT | FULL | 3.9e-10 |
| YKL222C_LalvinQA23 | START CODON | FULL | 3.9e-10 |
| YKL222C_PW5 | START CODON | FULL | 3.9e-10 |
| YKL222C_Vin13 | FRAMESHIFT | FULL | 3.9e-10 |
| YKL222C_VL3 | FRAMESHIFT | FULL | 3.9e-10 |
| YKL222C_ZTW1 | START CODON | FULL | 3.9e-10 |
| YKR064W_AWRI796 | FRAMESHIFT | FULL | 1.4e-13 |
| YKR064W_CBS7960 | FRAMESHIFT | FULL | 1.5e-13 |
| YKR064W_EC1118 | FRAMESHIFT | FULL | 1.4e-13 |
| YKR064W_FostersB | FRAMESHIFT | FULL | 1.4e-13 |
| YKR064W_FostersO | FRAMESHIFT | PARTIAL | 4.5e-11 |
| YKR064W_LalvinQA23 | FRAMESHIFT | FULL | 1.3e-13 |
| YKR064W_Vin13 | FRAMESHIFT | FULL | 1.4e-13 |
| YKR064W_VL3 | FRAMESHIFT | FULL | 1.4e-13 |
| YKR064W_YJM269 | FRAMESHIFT | FULL | 1.5e-13 |
| YLL054C_AWRI1631 | START CODON | FULL | 5.9e-12 |
| YLL054C_AWRI796 | FRAMESHIFT | FULL | 5.8e-12 |
| YLL054C_CLIB324 | FRAMESHIFT | FULL | 5.9e-12 |
| YLL054C_EC1118 | FRAMESHIFT | FULL | 5.8e-12 |
| YLL054C_FostersB | FRAMESHIFT | FULL | 5.9e-12 |
| YLL054C_FostersO | FRAMESHIFT | FULL | 5.9e-12 |

| | | | |
|---|---|---|---|
| YLL054C_LalvinQA23 | FRAMESHIFT | FULL | 5.9e-12 |
| YLL054C_PW5 | START CODON | FULL | 5.8e-12 |
| YLL054C_Vin13 | FRAMESHIFT | FULL | 5.9e-12 |
| YLL054C_VL3 | FRAMESHIFT | FULL | 5.8e-12 |
| YLL054C_ZTW1 | FRAMESHIFT | FULL | 6.9e-12 |
| YLR014C_AWRI1631 | FRAMESHIFT | FULL | 8.4e-15 |
| YLR014C_AWRI796 | FRAMESHIFT | FULL | 8.4e-15 |
| YLR014C_CLIB215 | FRAMESHIFT | FULL | 8.4e-15 |
| YLR014C_FostersB | FRAMESHIFT | FULL | 1.9e-14 |
| YLR014C_FostersO | FRAMESHIFT | FULL | 8.4e-15 |
| YLR014C_LalvinQA23 | FRAMESHIFT | FULL | 8.5e-15 |
| YLR014C_Vin13 | FRAMESHIFT | FULL | 8.4e-15 |
| YLR014C_VL3 | FRAMESHIFT | FULL | 8.4e-15 |
| YLR098C_AWRI796 | FRAMESHIFT | FULL | 7.7e-15 |
| YLR098C_FostersB | FRAMESHIFT | FULL | 7.7e-15 |
| YLR098C_FostersO | FRAMESHIFT | FULL | 7.7e-15 |
| YLR098C_LalvinQA23 | FRAMESHIFT | FULL | 7.7e-15 |
| YLR256W_FostersO | FRAMESHIFT | FULL | 1.9e-12 |
| YLR266C_AWRI796 | FRAMESHIFT | FULL | 1.2e-10 |
| YLR266C_FostersB | FRAMESHIFT | FULL | 1.1e-10 |
| YLR266C_FostersO | START CODON | FULL | 1.0e-10 |
| YLR266C_Vin13 | START CODON | FULL | 1.9e-08 |
| YLR266C_VL3 | FRAMESHIFT | FULL | 1.1e-10 |
| YLR278C_CBS7960 | Unknown | FULL | 8.8e-11 |
| YLR278C_CLIB215 | FRAMESHIFT | FULL | 8.7e-11 |
| YLR278C_FostersO | FRAMESHIFT | FULL | 8.7e-11 |
| YLR451W_LalvinQA23 | START CODON | FULL | 1.4e-12 |
| YML076C_AWRI796 | FRAMESHIFT | FULL | 2.1e-06 |
| YML076C_CLIB215 | START CODON | FULL | 1.8e-07 |
| YML076C_FostersB | FRAMESHIFT | FULL | 5.8e-06 |
| YML076C_FostersO | FRAMESHIFT | FULL | 2.1e-06 |
| YML076C_LalvinQA23 | FRAMESHIFT | FULL | 2.1e-06 |
| YMR019W_FostersO | FRAMESHIFT | FULL | 4.9e-12 |
| YMR019W_LalvinQA23 | FRAMESHIFT | FULL | 1.2e-12 |
| YMR019W_Vin13 | FRAMESHIFT | FULL | 2.5e-12 |
| YMR168C_AWRI1631 | FRAMESHIFT | FULL | 5.6e-13 |
| YMR280C_AWRI796 | FRAMESHIFT | FULL | 6.0e-15 |
| YMR280C_FostersB | FRAMESHIFT | FULL | 6.0e-15 |
| YMR280C_FostersO | FRAMESHIFT | FULL | 6.0e-15 |
| YMR280C_LalvinQA23 | FRAMESHIFT | FULL | 6.0e-15 |
| YMR280C_RedStar | FRAMESHIFT | FULL | 5.9e-15 |
| YMR280C_Vin13 | FRAMESHIFT | FULL | 6.0e-15 |
| YMR280C_YJM269 | FRAMESHIFT | FULL | 5.8e-15 |

| | | | |
|---|---|---|---|
| YNR063W_AWRI796 | FRAMESHIFT | FULL | 2.2e-14 |
| YNR063W_CLIB215 | FRAMESHIFT | FULL | 2.2e-14 |
| YNR063W_FostersO | FRAMESHIFT | FULL | 1.4e-14 |
| YNR063W_PW5 | FRAMESHIFT | FULL | 3.3e-14 |
| YNR063W_VL3 | FRAMESHIFT | FULL | 2.3e-14 |
| YOL089C_AWRI796 | FRAMESHIFT | FULL | 3.3e-13 |
| YOL089C_CBS7960 | FRAMESHIFT | FULL | 3.3e-13 |
| YOL089C_FostersB | FRAMESHIFT | FULL | 3.3e-13 |
| YOL089C_FostersO | FRAMESHIFT | FULL | 4.2e-13 |
| YOL089C_L1528 | START CODON | FULL | 3.3e-13 |
| YOL089C_LalvinQA23 | FRAMESHIFT | FULL | 3.3e-13 |
| YOL089C_PW5 | FRAMESHIFT | FULL | 3.3e-13 |
| YOL089C_Vin13 | FRAMESHIFT | FULL | 3.3e-13 |
| YOL089C_VL3 | FRAMESHIFT | FULL | 3.4e-13 |
| YOL089C_YJM789 | START CODON | FULL | 3.3e-13 |
| YOR162C_FostersB | FRAMESHIFT | FULL | 1.7e-12 |
| YOR162C_FostersO | FRAMESHIFT | FULL | 1.7e-12 |
| YOR162C_Vin13 | FRAMESHIFT | FULL | 1.7e-12 |
| YOR162C_VL3 | FRAMESHIFT | FULL | 1.7e-12 |
| YOR162C_ZTW1 | FRAMESHIFT | FULL | 2.4e-12 |
| YOR172W_AWRI796 | FRAMESHIFT | FULL | 8.8e-12 |
| YOR172W_JAY291 | START CODON | FULL | 8.9e-12 |
| YOR172W_Kyokai7 | START CODON | FULL | 5.1e-05 |
| YOR172W_LalvinQA23 | FRAMESHIFT | FULL | 8.8e-12 |
| YOR337W_AWRI796 | FRAMESHIFT | FULL | 3.5e-12 |
| YOR337W_FostersB | FRAMESHIFT | FULL | 3.5e-12 |
| YOR337W_FostersO | FRAMESHIFT | FULL | 3.5e-12 |
| YOR337W_LalvinQA23 | FRAMESHIFT | FULL | 3.7e-12 |
| YOR337W_T73 | FRAMESHIFT | FULL | 3.5e-12 |
| YOR337W_Vin13 | FRAMESHIFT | FULL | 3.6e-12 |
| YOR337W_VL3 | FRAMESHIFT | FULL | 3.7e-12 |
| YOR380W_AWRI796 | FRAMESHIFT | FULL | 2.2e-12 |
| YOR380W_LalvinQA23 | FRAMESHIFT | FULL | 2.2e-12 |
| YOR380W_VL3 | FRAMESHIFT | FULL | 2.2e-12 |
| YPL248C_AWRI1631 | FRAMESHIFT | FULL | 4.9e-14 |
| YPL248C_FostersB | FRAMESHIFT | FULL | 1.8e-13 |
| YPR196W_AWRI796 | FRAMESHIFT | FULL | 5.2e-12 |
| YPR196W_BY4741 | START CODON | FULL | 6.6e-12 |
| YPR196W_CLIB324 | FRAMESHIFT | FULL | 5.4e-12 |
| YPR196W_EC9-8 | FRAMESHIFT | FULL | 5.3e-12 |
| YPR196W_FostersB | FRAMESHIFT | FULL | 5.0e-12 |
| YPR196W_FostersO | FRAMESHIFT | PARTIAL | 4.2e-11 |
| YPR196W_LalvinQA23 | FRAMESHIFT | FULL | 5.3e-12 |

| | | | |
|---|---|---|---|
| YPR196W_SEY6210 | Unknown | FULL | 6.6e-12 |
| YPR196W_VL3 | FRAMESHIFT | FULL | 5.3e-12 |
