# Supplementary material for: CeGAL: Redefining a Widespread Fungal-Specific Transcription Factor Family Using an In Silico Error-Tracking Approach
Source: J Fungi (Basel). 2023 Mar 29;9(4):424. doi: 10.3390/jof9040424 (PMC10141177; doi:10.3390/jof9040424)
Supplement: Supplementary file 1 [file jof-09-00424-s001.zip › Table S4.pdf]

Table S4. Frequency of MHD domain pairs in the UniProtKB Swiss-Prot and TrEMBL sections. The numbers in brackets indicate the proportion of each domain pair with respect to the total number of MHD sequences.

	Swiss-Prot	TrEMBL	Total
Zn2C6-MHD	152 (89.4%)	80304 (63.4%)	80456 (63.4%)
C2H2-MHD	6 (3.5%)	6308 (5.0%)	6314 (5.0%)
MHD-only	12 (7.1%)	37634 (29.7%)	37646 (29.7%)
Other	0 (0%)	2445 (1.9%)	2445 (1.9%)
Total	170	126691	126861
