# Supplementary material for: CeGAL: Redefining a Widespread Fungal-Specific Transcription Factor Family Using an In Silico Error-Tracking Approach
Source: J Fungi (Basel). 2023 Mar 29;9(4):424. doi: 10.3390/jof9040424 (PMC10141177; doi:10.3390/jof9040424)
Supplement: Supplementary file 1 [file jof-09-00424-s001.zip › Table S5.pdf]

Table S5. Swiss-Prot entries for 12 MHD-only proteins, and cross-references to genome and literature databases.

Swiss-Prot ID	Organism	Function	Swiss-Prot status	ENSEMBL gene ID / Genbank ID	PUBMED ID
S0DS79	*Gibberella fujikuroi*	Equisetin cluster transcription factor	Experimental evidence at transcript level	CCT65290	28379186
S4W775	*Fusarium heterosporum*	Equisetin cluster transcription factor	Protein predicted	KC439347	23614392
B6HNK4	*Penicillium rubens*	Sorbicillinoid biosynthetic cluster transcription factor 2	Protein predicted	CAP95406	28618182
Q5SBL4	*Fusarium heterosporum*	A cluster transcription factor fsdR	Protein predicted	AY700570	23614392
Q5AR44	*Aspergillus nidulans*	Transcription factor asqA	Protein predicted	CBF82261	25251934
D7PI12	*Penicillium aethiopicum*	Probable transcription factor gsfR1	Experimental evidence at protein level	GU574478.1	20534346
O59746	*Schizosaccharomyces pombe*	Uncharacterized transcriptional regulatory protein	Experimental evidence at protein level	SPBC530.11c	
Q9P7H9	*Schizosaccharomyces pombe*	Uncharacterized transcriptional regulatory protein	Protein inferred from homology	SPAC105.03c.1	
A0A5C1RF03	*Didymella fabae*	Ascochitine biosynthesis cluster transcriptional regulator	Protein inferred from homology	MN052632.1	31554725
D7UQ41	*Alternaria solani*	Probable transcription factor sol4	Protein predicted	AB514562	20486243
B8NJG5	*Aspergillus flavus*	Probable transcription factor lepB	Protein predicted	EED49864	20447271, 26051490
A6SSW6	*Botryotinia fuckeliana*	Transcription factor BOA15	Protein predicted	FR718884	21722295
