# Supplementary material for: CeGAL: Redefining a Widespread Fungal-Specific Transcription Factor Family Using an In Silico Error-Tracking Approach
Source: J Fungi (Basel). 2023 Mar 29;9(4):424. doi: 10.3390/jof9040424 (PMC10141177; doi:10.3390/jof9040424)
Supplement: Supplementary file 1 [file jof-09-00424-s001.zip › Table S6.pdf]

Table S6. Number of MHD-containing sequences with different domain combinations in public databases: Swiss-Prot and TrEMBL (sequences mapped to ENSEMBL only) and number of DBD identified by our error identification protocol.

	Swiss-Prot		
	Database	DBD identified by protocol	After correction
Zn2C6-MHD	152 (89.4%)	7 (58.3%)	159 (93.5%)
C2H2-MHD	6 (3.5%)	1 (8.3%)	7 (4.1%)
MHD-only	12 (7.1%)	4 (33.3%)	4 (2.4%)
	TrEMBL		
	Database	DBD identified by protocol	After correction
Zn2C6-MHD	36875 (65.4%)	13734 (81.9%)	50609 (89.7%)
C2H2-MHD	2763 (4.9%)	748 (4.5%)	3511 (6.2%)
MHD-only	16760 (29.7%)	2278 (13.6%)	2278 (4.0%)
