# Supplementary material for: CeGAL: Redefining a Widespread Fungal-Specific Transcription Factor Family Using an In Silico Error-Tracking Approach
Source: J Fungi (Basel). 2023 Mar 29;9(4):424. doi: 10.3390/jof9040424 (PMC10141177; doi:10.3390/jof9040424)
Supplement: Supplementary file 1 [file jof-09-00424-s001.zip › Table S7.pdf]

Table S7. Comparison of MHD-containing sequences from Ascomycota and Basidiomycota. Number of MHD-containing sequences with different domain combinations in public databases: Uniprot (sequences mapped to ENSEMBL only) and number of DBD identified by our error identification protocol.

Ascomycota			
	Uniprot	DBD identified by protocol	After correction
Zn2C6-MHD	32324 (66.8%)	11221 (84.1%)	43545 (90.0%)
C2H2-MHD	2715 (5.6%)	638 (4.8%)	3353 (6.9%)
MHD-only	13338 (27.6%)	1479 (11.1%)	1479 (3.1%)
Basidiomycota			
	Uniprot	DBD identified by protocol	After correction
Zn2C6-MHD	3249 (72.8%)	859 (77.9%)	4108 (92.1%)
C2H2-MHD	111 (2.5%)	14 (1.3%)	1125 (2.8%)
MHD-only	1102 (24.7%)	229 (20.8%)	229 (5.1%)
