# Supplementary figures and images for: CeGAL: Redefining a Widespread Fungal-Specific Transcription Factor Family Using an In Silico Error-Tracking Approach
Source: J Fungi (Basel). 2023 Mar 29;9(4):424. doi: 10.3390/jof9040424 (PMC10141177; doi:10.3390/jof9040424)

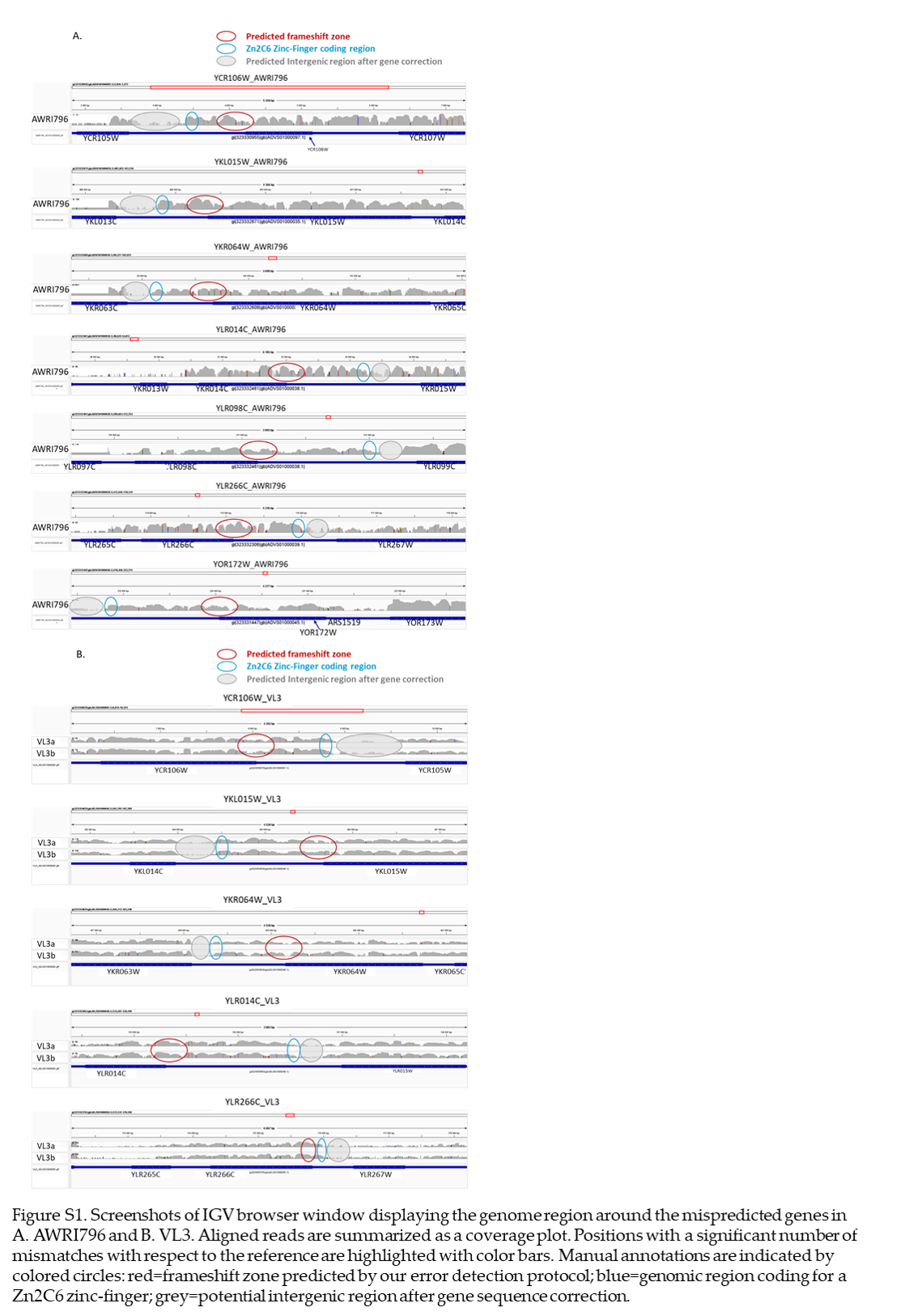

Supplement: Supplementary file 1 [file jof-09-00424-s001.zip › Figure S1.png]

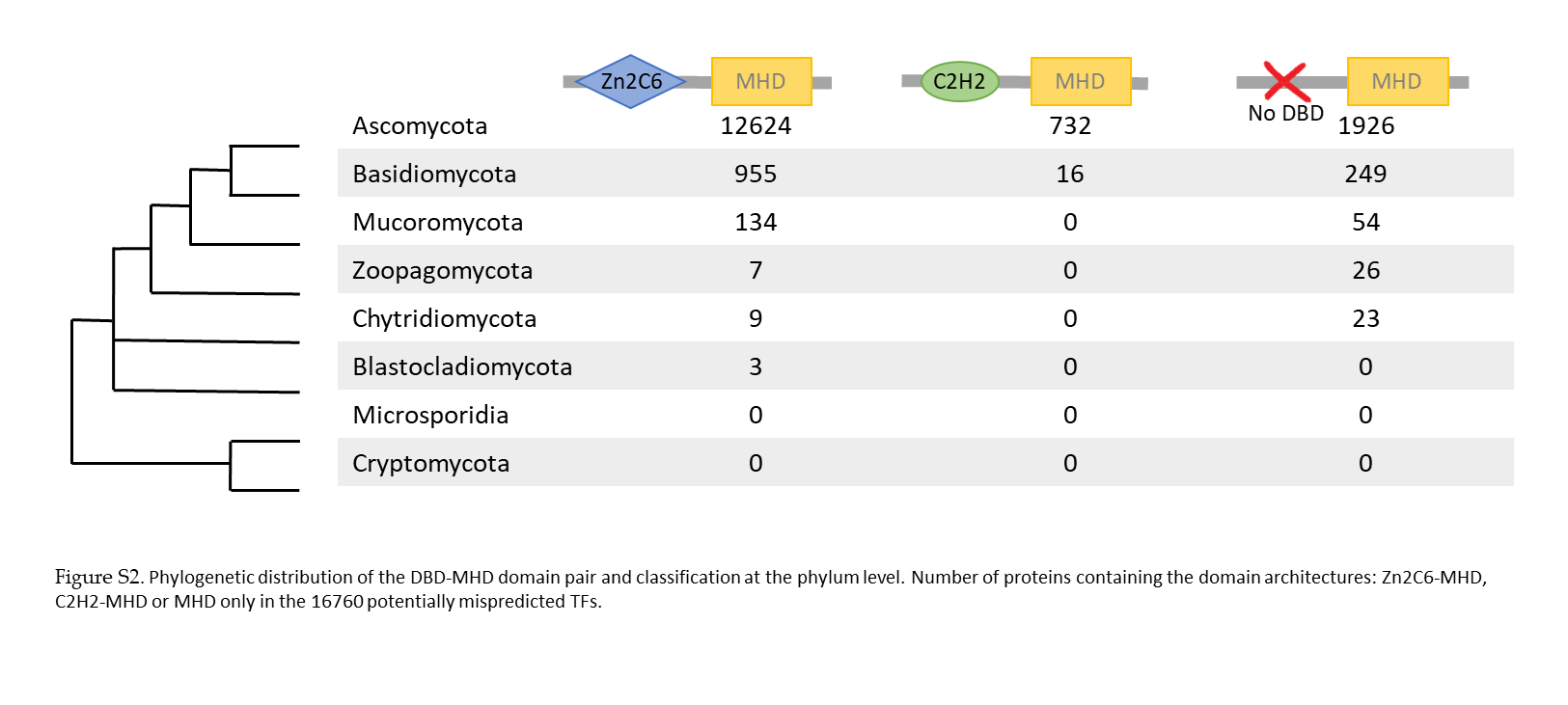

Supplement: Supplementary file 1 [file jof-09-00424-s001.zip › Figure S2.png]
